# Rhinovirus Species in Acute Respiratory Tract Infections and the Risk of Asthma in Children

**DOI:** 10.1002/ppul.71782

**Published:** 2026-08-10

**Authors:** Ville Forsström, Matti Waris, Ville Peltola, Laura Toivonen

**Affiliations:** ^1^ Department of Paediatrics and Adolescent Medicine Turku University Hospital and University of Turku Turku Finland; ^2^ Virology Unit, Institute of Biomedicine University of Turku Turku Finland

**Keywords:** acute respiratory infection, asthma, childhood asthma, children, rhinovirus species, upper respiratory tract infection, wheezing

## Abstract

**Background:**

Rhinoviruses (RV) are the most common pathogens causing respiratory tract infections, and early childhood RV wheezing illnesses are a known risk factor for childhood asthma. The role of acute upper respiratory tract infections (URTI) caused by different RV species in asthma development is not well known.

**Methods:**

We conducted a nested case‐control study in a birth cohort of 923 children followed prospectively from birth to age 7 years. Nasal swab samples collected during acute respiratory tract infections (ARI) at age 0–24 months were analyzed by PCR, and RV‐positive samples were genotyped. Each case with asthma at age 7 years was matched with three cohort children without asthma. Conditional logistic regression was used to test associations between RV wheezing illnesses or RV URTIs and asthma risk.

**Results:**

We identified 56 cases and 168 matched controls. In total, 807 RV‐positive samples were successfully classified. Wheezing illnesses caused by RV‐A and RV‐C at age 0–24 months were associated with an increased risk of asthma at age 7 years (for RV‐A, odds ratio [OR]: 1.60, 95% confidence interval [CI]: 1.05–2.43, *p* = 0.03; for RV‐C, OR: 1.66, 95% CI: 1.11–2.49, *p* = 0.01). RV‐A, RV‐B, or RV‐C positive URTIs were not associated with asthma risk.

**Conclusion:**

Both RV‐A and RV‐C ‐induced early wheezing illnesses were associated with an increased risk of childhood asthma. Acute URTIs caused by RV‐A, RV‐B, or RV‐C were not associated with asthma risk.

## 1 Introduction

Asthma is the most prevalent chronic disease in children globally [1]. Known risk factors for childhood asthma include host risk factors such as atopy and parental asthma and environmental risk factors such as exposure to tobacco smoke and reduced microbial exposure [2, 3, 4, 5]. Early childhood wheezing illness is a strong predictor for the later development of asthma, and both respiratory syncytial virus (RSV) and rhinovirus (RV) induced wheezing episodes are associated with the increased risk of childhood asthma [6, 7, 8, 9].

The most typical respiratory infection in children is not wheezing illness, however, but the common cold [10]. The association between upper respiratory tract infections (URTIs), such as the common cold, and childhood asthma is less well understood. We have previously reported that the high rate of acute respiratory infections (ARI) at first years of life is associated with an increased risk of childhood asthma [11]. As rhinoviruses are the most common pathogens causing common colds and a major factor in asthma exacerbations [10, 12], further investigation of the role of different RV species in the development of asthma is warranted.

RVs are single‐stranded RNA viruses belonging in the *Enterovirus* genus in the family of Picornaviruses. They can be further classified into three species, RV A, RV B, and RV C, based on their genetic differences [13, 14]. More than 160 different RV types belonging to species A, B, and C have been documented [15]. Recent advances in sequencing technology have made large‐scale typing of rhinoviruses possible. This has led to findings that RV species A and C are more likely to cause bronchiolitis while RV‐B infections are more likely to be asymptomatic or cause only mild symptoms [16, 17, 18]. RV‐A and RV‐C are also the predominant rhinovirus species detected during acute respiratory infections and asymptomatic state [19]. Earlier research has indicated that children with RV‐C wheezing illness have an elevated risk of recurrent wheezing [20]. This correlation may be attributed to Cadherin‐related family member 3 (CDHR3) protein, which serves as a receptor for RV‐C. Notably, a single nucleotide polymorphism (SNP) in the *CDHR3* gene has been linked to an increased susceptibility to RV‐C respiratory illnesses in general [21, 22] and childhood asthma [23].

To our knowledge, no prior studies of the associations of non‐wheezing ARIs caused by different RV species with later childhood asthma have been conducted. To address this knowledge gap, we conducted a nested case‐control study and analyzed the association between early ARIs with different RV species and the risk of childhood asthma at age 7 years.

## 2 Materials and Methods

### 2.1 Study Design and Population

This nested case‐control study was conducted within the prospective, population‐based birth cohort study called the Steps to the Healthy Development and Well‐being of Children (STEPS Study), where children born between 2008 and 2010 in the Hospital District of Southwest Finland are followed from birth to adulthood. The children were enrolled at birth in an intensive follow‐up for acute respiratory tract infections (ARI) as described earlier [11, 24, 25]. No selection criteria other than language (Finnish or Swedish speaking family) were applied to recruiting the families in the STEPS Study. The children were followed for ARIs with daily symptom diaries and study clinic visits from birth to 2 years of age. Parents were encouraged to take the children to the study clinic if they observed symptoms of ARI. Nasal swab samples were collected during ARIs either at the study clinic by a physician or at home by parents who sent the swabs to the study clinic as described earlier [24]. Data on outpatient and emergency department visits at hospitals, hospitalizations from birth to 2 years of age, and physician‐diagnosis of asthma at age 6.5–7.5 years were retrieved from medical records of the Hospital District of Southwest Finland [11, 26]. The use of asthma medication at age 6.5–7.5 years was retrieved from electronic prescription records. Nasal swab specimens were collected by study personnel using flocked nylon swabs (Copan, Brescia, Italy) at scheduled participant visits at age 2, 13 and 24 months during healthy state. Background data were collected from the National Birth Registry and by structured questionnaires.

The STEPS Study was approved by the Ethics Committee of the Hospital District of Southwest Finland (approval number 16/180/2008). Parents of the participating children gave their written, informed consent. The study was carried out in accordance with the Declaration of Helsinki.

### 2.2 Definitions and Matching

Acute respiratory tract infection (ARI) was defined as an episode of rhinitis or cough, with or without fever or wheezing, documented in the symptom diary by parents or as any physician‐diagnosed ARI documented in the study clinic visits or in the electronic medical records [26]. Wheezing illness was defined as symptoms of wheezing documented in the symptom diary by the parents or diagnosed by a physician (bronchiolitis, recurrent wheezing, or acute exacerbation of asthma [11]). Upper respiratory tract infection (URTI) was defined as an ARI without recorded wheezing illness or pneumonia.

Cases were defined as children with physician‐diagnosed asthma at age 7 years. Physician‐diagnosed asthma was defined as a diagnosis of asthma in the medical records at age 6.5–7.5 years (age 7 years) with or without an electronic prescription of inhaled corticosteroids for asthma at the same age [11, 27]. We matched each case with 3 control children within the cohort (no asthma during age 6.5–7.5 years) using R package Epi matching on sex, birth year (2008, 2009, 2010), and birth season (4 seasons). Children missing registry data on asthma at age 6.5–7.5 years, or with ARI follow‐up of less than 12 months were excluded.

### 2.3 Respiratory Virus Detection and Typing

First, nucleic acids were extracted from the nasal swabs collected during ARIs using NucliSense easyMag (BioMerieux, Boxtel, The Netherlands) or MagnaPure 96 (Roche, Penzberg, Germany). RV, RSV, enteroviruses, and influenza A and B viruses were analyzed using PCR as described earlier [28, 29, 30]. Rhinovirus‐positive samples were then sequenced and classified in species A, B, and C based on sequencing of a genomic VP4/2 region after amplification with nested PCR primers [31]. The sequences were analyzed using Basic Local Alignment Search Tool (https://blast.ncbi.nlm.nih.gov) and types and species were assigned according to the proposed principles [13].

### 2.4 Statistical Analysis

Associations between rhinovirus positive URTIs and wheezing illnesses and asthma at age 7 years were analyzed using conditional logistic regression analysis and adjusted for parental asthma. Separate models were used for RV‐A, RV‐B and RV‐C positive infections. Number of RV infections was used as a continuous variable. Two‐tailed P‐values were reported, with *p* < 0.05 considered statistically significant. The data were analyzed using IBM SPSS Statistics software version 27 and R version 4.2.2.

## 3 Results

### 3.1 Study Population

**Figure 1:**
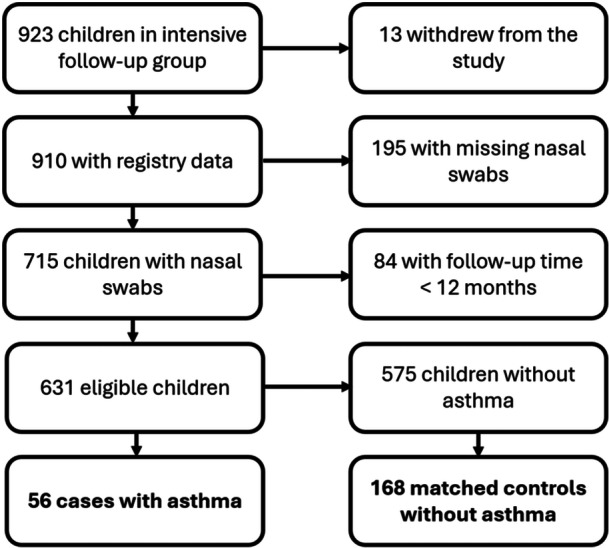
Formation of the study cohort.

Of the 923 children recruited in the STEPS study, 13 children withdrew from the study, and registry data on asthma at age 7 years was available for 910 (99%) children. Of these children, 715 (77.5%) had nasal swabs collected at 2 and 13 months of age and during ARIs. Children with an ARI follow‐up < 12 months (*n* = 84) were excluded from the analysis. Of the children, 56 (7.8%) were diagnosed with asthma at age 7 years and identified as cases. The matching was performed in the cohort of 631 children (56 cases with asthma and 575 children without asthma at age 7 years). Children with asthma were successfully matched with 168 controls without asthma. The formation of the study cohort is presented in Figure 1. The baseline characteristics of cases and controls are presented in Table 1. Parental asthma was more frequent in children with asthma than in controls without asthma.

**Table 1:** Baseline characteristics of the study children.

	Children with asthma at age 7 years (Cases, *n* = 56)	Children without asthma at age 7 years (Controls, *n* = 168)	*p* value
Female	20 (36%)	60 (36%)	1.00
Delivery by *c*‐section	12 (21%)	20 (12%)	0.12
Premature (< 37 weeks)	3 (5%)	6 (4%)	0.69
Older siblings	23 (41%)	72 (43%)	0.88
Parental asthma	18 (32%)	15 (9%)	**< 0.01**
Parental allergy	26 (47%)	80 (48%)	1.00
Breastfeeding at age 6 mo	28 (56%)	101 (65%)	0.31
Parental smoking	13 (28%)	22 (17%)	0.13
Daycare attendance at age 13 mo	8 (15%)	40 (24%)	0.19

*Note:* Data are presented as no (%). Percentages may not equal 100 because of missingness.

### 3.2 RV Species and Acute Respiratory Infections

A total of 1916 nasal swab samples were collected from the 224 children included in this nested case‐control study (median 6 per child, interquartile range [IQR]: 3–9). Of the samples 1342 (70.0%) were collected during ARIs and 574 (30.0%) during routine follow‐up visits at healthy state. RV was detected in 870 (45.4%) of the samples (median 3 per child, IQR: 2–6). Of these samples, 807 (92.8%) were successfully classified in species RV‐A, RV‐B, and RV‐C based on their VP4/2 sequence. A total of 248 RV‐A, 28 RV‐B, and 531 RV‐C positive samples were detected.

Of the total of 1342 ARIs, 792 (59.0%) were RV‐positive. The distribution of the ARIs and RV species is presented in Table 2. Of the RV‐positive ARIs, 687 (86.7%) were URTIs, and 105 (13.3%) were wheezing illnesses. Six RV‐positive wheezing illnesses led to hospital admission. RV species were successfully classified in 646 (94.0%) of the samples collected during URTIs and 95 (90.5%) of the samples collected during wheezing illnesses. RV‐C was detected in 36.9% of the ARIs, RV‐A in 17.2%, and RV‐B in 1.1% (Table 2). RV‐C was detected in 38.6% of the URTIs and RV‐A in 16.5%. RV‐C was detected in 26.9% of the wheezing illnesses and RV‐A in 21.3%. There were no recorded RV‐B positive wheezing illnesses.

**Table 2:** Rhinovirus species by clinical presentation of respiratory infections at age 0–24 months.

RV species	All ARIs	Wheezing illnesses	URTIs	Samples at scheduled follow‐up visits†
	*n* = 1342	*n* = 197	*n* = 1145	*n* = 574
RV‐negative	550 (41.0%)	92 (46.7%)	458 (40.0%)	497 (86.6%)
RV‐positive	792 (59.0%)	105 (53.3%)	687 (60.0%)	77 (13.4%)
RV‐A	231 (17.2%)	42 (21.3%)	189 (16.5%)	17 (3.0%)
RV‐B	15 (1.1%)	0 (0.0%)	15 (1.3%)	13 (2.3%)
RV‐C	495 (36.9%)	53 (26.9%)	442 (38.6%)	36 (6.3%)

*Note:* Percentages may not equal 100 because of missingness.

Abbreviations: ARI, acute respiratory infection; RV, rhinovirus; URTI, upper respiratory tract infection.

^†^ Children at scheduled follow‐up visits at age 2, 13 and 24 months.

RV was detected in 15 (7.4%) of the children at age 2‐month healthy follow‐up visit, in 20 (10.8%) at age 13, and in 42 (22.6%) of the children at age 24‐month healthy follow‐up visit. RV‐C was detected in 36 (6.3%), RV‐A in 17 (3.0%), and RV‐B in 13 (2.3%) children at healthy state (Table 2). The infection burden of RV‐A, RV‐B and RV‐C positive URTIs and wheezing illnesses is presented in Table S1.

### 3.3 RV‐Positive Acute Respiratory Infections and Risk of Asthma

Wheezing illnesses caused by RV‐A and RV‐C at age 0‐24 months were associated with a higher risk of childhood asthma at age 7 years (for RV‐A, odds ratio [OR]: 1.60, 95% confidence interval [CI]: 1.05–2.43, *p* = 0.03 and for RV‐C, OR: 1.66, 95% CI: 1.11–2.49, *p* = 0.01). URTIs caused by RV‐A, RV‐B, or RV‐C were not associated with a risk of childhood asthma (Table 3).

**Table 3:** Rhinovirus infections at age 0–24 months by species and clinical presentation and the risk of asthma at age 7 years, adjusted for parental asthma.

	Risk of asthma at age 7 years	
	OR (95% CI)	*p* value
Wheezing Illnesses at age 0–24 months		
RV‐A	**1.60 (1.05–2.43)**	**0.03**
RV‐B^a^	N/A	
RV‐C	**1.66 (1.11‐2.49)**	**0.01**
URTIs at age 0–24 months		
RV‐A	0.90 (0.67–1.19)	0.45
RV‐B	0.56 (0.13–2.34)	0.42
RV‐C	0.92 (0.78–1.08)	0.28

Abbreviations: CI, confidence interval; N/A, not applicable; OR, odds ratio; RV, rhinovirus; URTI, upper respiratory tract infection.

*Note:* The number of RV infections was used as a continuous variable. Change in odds for asthma per each infection.

^a^ No RV‐B wheezing illnesses were documented.

### 3.4 Etiology of First Wheezing Illness and First URTI, and Risk of Asthma

In this matched case‐control study, 118 (52.6%) children had a wheezing illness during the follow‐up period. In 83 (70.3%) of the first wheezing illnesses, a viral sample was available. Of these, 42 were RV‐negative, 13 positive for RV‐A, and 24 positive for RV‐C. The etiology of the first wheezing illness was not significantly associated with risk of asthma, although RV‐A etiology of first wheezing illness was at the limit of statistical significance when compared to children with no wheezing (OR: 2.68, 95% CI: 0.99–7.27, *p* = 0.05). Children with no RV detected during their first wheezing illness had also an increased risk of asthma (OR: 1.96, 95% CI: 1.05–3.66, *p* = 0.03, Table 4). RV species of the first URTI was not associated with the risk of childhood asthma when compared to RV‐negative URTIs (Table 4).

**Table 4:** Etiology of the first URTI and first wheezing illness and the risk of asthma at age 7 years, adjusted for parental asthma.

	Risk of asthma at age 7 years	
	OR (95% CI)	*p* value
Etiology of the first wheezing illness		
No wheezing (*n* = 106)	ref	
Wheezing, RV negative or not studied (*n* = 77)	**1.96 (1.05–3.66)**	**0.03**
RV‐A (*n* = 13)	2.68 (0.99–7.27)	0.05
RV‐B^a^	N/A	
RV‐C (*n* = 24)	1.74 (0.70‐4.33)	0.24
Etiology of the first URTI		
RV‐negative (*n* = 50)^b^	ref	
RV‐A (*n* = 18)	2.14 (0.62–7.35)	0.23
RV‐B (*n* = 2)	2.06 (0.21–20.40)	0.54
RV‐C (*n* = 50)	0.86 (0.34–2.22)	0.76

Abbreviations: CI, confidence interval; N/A, not applicable; OR, odds ratio; RV, rhinovirus; URTI, upper respiratory tract infection.

^a^ No RV‐B wheezing illnesses were documented.

^b^ Two children had no documented URTIs and were not included as a reference group.

## 4 Discussion

In this nested case‐control study conducted within a population‐based birth‐cohort, we found that RV‐A and RV‐C wheezing illnesses were associated with an increased risk of asthma. However, URTIs caused by any RV species were not associated with asthma risk. In line with these findings, we have earlier reported that RV wheezing illnesses were associated with a higher risk of childhood asthma while RV‐positive ARIs were not [11]. This further analysis using RV genotyping complements our earlier findings by showing that there was no association between URTIs and asthma risk regardless of the RV species, whereas wheezing illnesses of both RV‐A and RV‐C etiology were associated with a higher risk of childhood asthma.

Wheezing illnesses are a known predictor for childhood asthma [6, 7, 8]. Both RSV‐ and RV‐induced wheezing illnesses are associated with a higher risk for later childhood asthma, but the causative agent's role in the development of asthma is debated [16, 32]. RV‐A and RV‐C cause more often bronchiolitis and asthma exacerbations than RV‐B, which presents generally with milder symptoms [16, 17, 18, 33]. In a previous study, both RV‐A and RV‐C induced bronchiolitis leading to hospitalization were associated with an increased asthma risk, but further research in unselected populations is warranted [33]. The findings of our study suggest that while wheezing itself is a major risk factor for asthma, the causative pathogen and even its subtype (i.e, RV species) may also play a role in the development of asthma. Previous studies of the associations between wheezing illnesses and childhood asthma have for the most part included children hospitalized for a wheezing illness [6, 9]. One of the strengths of this study is that in this population‐based birth‐cohort, we were also able to record outpatient treated wheezing illnesses and wheezing illnesses not requiring contact to healthcare providers. Despite the milder clinical presentation of the wheezing illnesses, the association with asthma was clear, widening the generalizability of our findings compared to earlier studies.

Previously, we and others have reported that ARIs are associated with an increased risk of developing asthma [11, 34]. Strongest effect has been reported between lower respiratory infections and an increased asthma risk. A European meta‐analysis in > 150,000 children showed that lower respiratory infections were associated with school‐age asthma and lower forced expiratory volume in 1 s (FEV1) and FEV1/FEV in school age [34]. In our previous study, we reported that while a higher number of URTIs was associated with asthma risk, URTIs caused by RV were not [11]. Functional differences between the RV species make it plausible, however, that the associations between URTIs and asthma risk could differ between the RV species. The most notable of the differences between RV species is in the entry of the viruses to the target cell. All RV‐B and most of RV‐A types interact with intercellular adhesion molecule 1 and some RV‐A types with low density lipoprotein receptor family members, whereas RV‐C interacts with CDHR3 [15]. To our knowledge, associations between RV species in non‐wheezing infections and later asthma have previously been studied only in retrospective settings with serologic tests [35]. In our study, we demonstrated that URTIs attributed to different RV species were not associated with increased asthma risk. This finding is interesting, as it suggests that the correlation between RV infections and the asthma risk does not have a uniform pattern across the spectrum of clinical infections.

Early childhood is a time for rapid development of the airway microbiome and the immune system with complex, often bidirectional interactions. The activation of the innate immune system and remodeling of the immune responses affects microbiome and susceptibility to pathogens, and vice versa [36]. For example, RV‐C infection in infants hospitalized for bronchiolitis was shown to associate with *Moraxella*‐dominant microbial profile, while RV‐A was associated with *Haemophilus* and RSV with *Streptococcus* dominance [37]. Furthermore, RV infection is reported to facilitate acquisition and within‐family transmission of *S. pneumoniae* [31]. Early life environmental exposures can also modify the host response to various allergens and pathogens. A study conducted in Wisconsin showed that children living in a farm environment had a lower incidence of allergic rhinitis, eczema, and severe respiratory illnesses [38]. Similar results have been reported from Europe [39]. It is therefore plausible that the first ARIs could influence the development of the immune system and predispose to a higher asthma risk. Rhinovirus etiology of the first wheezing illness has previously been reported to predict atopic asthma [40]. The role of the rhinovirus species involved in the first URTI and in the first wheezing illness in asthma development remains unclear. In this study, we found that no single RV species causing the first URTI or first wheezing illness was significantly associated with asthma risk.

The role of RV‐C infections in the development of asthma is especially interesting, as a SNP rs6967330 in *CDHR3* gene is associated with childhood asthma, asthma exacerbations, and wheezing illnesses [21, 23]. The SNP regulates CDHR3 surface protein expression on the epithelial cells of the airways, which acts as a RV‐C receptor and increases the binding and replication of RV‐C in epithelial cells [16]. Earlier studies have shown the SNP to associate with an increased risk for RV‐C infections [21]. This was also shown in our earlier study where *CDHR3* asthma risk allele was associated with a higher risk of RV‐positive non‐wheezing ARIs while not being significantly associated with the risk of RV‐positive wheezing illnesses [41]. Bergroth and colleagues have previously reported that RV‐C positive severe bronchiolitis is associated with earlier and prolonged use of asthma medication [32]. Considering these findings, it would be reasonable to infer that RV‐C infections could be associated with a higher risk for childhood asthma. In contrast to previous studies, in our study, the RV‐C etiology of the first wheezing illness was not significantly associated with asthma risk. Furthermore, in the present study RV‐C URTIs were not associated with risk of childhood asthma.

There are potential limitations to this study. First, the number of wheezing illnesses of specific etiologies was limited, reducing the power to detect weaker associations. For example, there were no recorded wheezing illnesses of RV‐B etiology. Second, the study was conducted in Finnish children, which is to be considered when generalizing the results to other populations. However, we conducted this study in an unselected birth‐cohort and included all wheezing illnesses, including outpatient and home‐treated wheezing illnesses, which improves the internal validity and generalizability of our findings. Third, potential detection bias should be noted, as children with more symptoms or healthcare contacts may be more likely to have respiratory samples obtained. However, this risk was reduced by the study design, as nasal samples were also collected at home by parents at the onset of respiratory symptoms.

## 5 Conclusion

In this study, URTIs caused by RV species A, B, or C were not associated with risk of asthma. Both RV‐A and RV‐C ‐induced early wheezing illnesses were associated with an increased risk of childhood asthma, while the RV species of the first URTI or wheezing illness was not significantly associated with asthma risk.

## Author Contributions

**Ville Forsström:** conceptualization, methodology, formal analysis, writing – review and editing, writing – original draft. **Matti Waris:** conceptualization, methodology, investigation, supervision, writing – original draft, writing – review and editing. **Ville Peltola:** writing – original draft, writing – review and editing, conceptualization, methodology, supervision, resources, investigation, funding acquisition. **Laura Toivonen:** conceptualization, methodology, investigation, data curation; supervision, resources, writing – review and editing, writing – original draft, funding acquisition, formal analysis.

## Conflicts of Interest

The authors declare no conflicts of interest.

## Supporting information


Supporting File

## Data Availability

The data that support the findings of this study are available from the corresponding author upon reasonable request.
