# Supplementary material for: Rhinovirus Species in Acute Respiratory Tract Infections and the Risk of Asthma in Children
Source: Pediatr Pulmonol. 2026 Aug 10;61(8):e71782. doi: 10.1002/ppul.71782 (PMC13454847; doi:10.1002/ppul.71782)
Supplement: Supplementary file 1 [file PPUL-61-0-s001.docx]

**Supplementary material**

**Rhinovirus Species in Acute Respiratory Tract Infections and the Risk of Asthma in Children**

Ville Forsström, MD, PhD^1^, Matti Waris, PhD^2^, Ville Peltola, MD, PhD^1^, and Laura Toivonen, MD, PhD^1^

^1^ Department of Paediatrics and Adolescent Medicine, Turku University Hospital and University of Turku, 20520 Turku, Finland
^2^ Virology Unit, Institute of Biomedicine, University of Turku, 20520 Turku, Finland

**Supplementary Table 1. Infection burden of RV-A, RV-B or RV-C positive URTIs and wheezing illnesses at age 0-24 months.**

|  | Children with at least one infection (%) (n = 224) | Median per child (IQR) |
| --- | --- | --- |
| **URTI** |  |  |
| RV-A | 119 (53.1) | 1 (0-1) |
| RV-B | 14 (6.3) | 0 (0-0) |
| RV-C | 170 (75.9) | 1 (1-3) |
|  |  |  |
| **Wheezing illness** |  |  |
| RV-A | 34 (15.2) | 0 (0-0) |
| RV-B | 0 (0.0) | 0 (0-0) |
| RV-C | 42 (18.8) | 0 (0-0) |

RV, rhinovirus; URTI, upper respiratory tract infection; IQR, interquartile range.
